# Uncovering Plaque Erosion: A Distinct Pathway in Acute Coronary Syndromes and a Gateway to Personalized Therapy

**DOI:** 10.3390/jcm14155456

**Published:** 2025-08-03

**Authors:** Angela Buonpane, Alberto Ranieri De Caterina, Giancarlo Trimarchi, Fausto Pizzino, Marco Ciardetti, Michele Alessandro Coceani, Augusto Esposito, Luigi Emilio Pastormerlo, Angelo Monteleone, Alberto Clemente, Umberto Paradossi, Sergio Berti, Antonio Maria Leone, Carlo Trani, Giovanna Liuzzo, Francesco Burzotta, Filippo Crea

**Affiliations:** 1 Center of Excellence for Cardiovascular Sciences, Ospedale Isola Tiberina-Gemelli Isola, 00186 Rome, Italy; antoniomaria.leone@fbf-isola.it (A.M.L.); filippo.crea@fbf-isola.it (F.C.); 2 Fondazione Toscana G. Monasterio, Ospedale del Cuore G, Pasquinucci, 54100 Massa, Italy; adecaterina@ftgm.it (A.R.D.C.); fpizzino@ftgm.it (F.P.); mciard@ftgm.it (M.C.); michecoc@ftgm.it (M.A.C.); aesposito@monasterio.it (A.E.); lpastor@ftgm.it (L.E.P.); uparadossi@ftgm.it (U.P.); ifcberti@ftgm.it (S.B.); 3 Interdisciplinary Center for Health Sciences, Scuola Superiore Sant’Anna, 56100 Pisa, Italy; 4 Department of Radiology, Fondazione G. Monasterio CNR-Regione Toscana, 56124 Pisa, Italy; monteleone@ftgm.it (A.M.); clemente@ftgm.it (A.C.); 5 Department of Cardiovascular Sciences, Fondazione Policlinico Universitario A. Gemelli IRCCS, Università Cattolica Sacro Cuore, Rome, Largo Agostino Gemelli, 0168 Rome, Italy; carlo.trani@unicatt.it (C.T.); giovanna.liuzzo@unicatt.it (G.L.); francesco.burzotta@unicatt.it (F.B.)

**Keywords:** plaque erosion, acute coronary syndromes, optical coherence tomography, personalized care

## Abstract

Plaque erosion (PE) is now recognized as a common and clinically significant cause of acute coronary syndromes (ACSs), accounting for up to 40% of cases. Unlike plaque rupture (PR), PE involves superficial endothelial loss over an intact fibrous cap and occurs in a low-inflammatory setting, typically affecting younger patients, women, and smokers with fewer traditional risk factors. The growing recognition of PE has been driven by high-resolution intracoronary imaging, particularly optical coherence tomography (OCT), which enables in vivo differentiation from PR. Identifying PE with OCT has opened the door to personalized treatment strategies, as explored in recent trials evaluating the safety of deferring stent implantation in selected cases in favor of intensive medical therapy. Given its unexpectedly high prevalence, PE is now recognized as a common pathophysiological mechanism in ACS, rather than a rare exception. This growing awareness underscores the importance of its accurate identification through OCT in clinical practice. Early recognition and a deeper understanding of PE are essential steps toward the implementation of precision medicine, allowing clinicians to move beyond “one-size-fits-all” models toward “mechanism-based” therapeutic strategies. This narrative review aims to offer an integrated overview of PE, tracing its epidemiology, elucidating the molecular and pathophysiological mechanisms involved, outlining its clinical presentations, and placing particular emphasis on diagnostic strategies with OCT, while also discussing emerging therapeutic approaches and future directions for personalized cardiovascular care.

## 1. Introduction

*“We shape our tools and thereafter our tools shape us”. Marshall McLuhan (Understanding Media: The Extensions of Man, 1964)*

Acute coronary syndromes (ACSs) have classically been attributed to plaque rupture (PR), wherein a fibrous cap breaks and triggers intracoronary thrombosis. However, plaque erosion (PE), the superficial denudation of endothelial cells without a fibrous cap rupture, has emerged from a once-rare finding to a common underlying mechanism of ACS. Early autopsy series focusing on fatal myocardial infarctions (MIs) reported PE in only ~20% of cases. With advances in intracoronary imaging like optical coherence tomography (OCT), contemporary studies now recognize PE in roughly 30–40% of ACS patients. This paradigm shift in understanding is significant: PE is no longer overshadowed by rupture but rather is appreciated as a distinct mechanism accounting for more than one-third of acute coronary events. Recognition of PE prevalence has prompted new research and tailored therapeutic approaches in ACS. Importantly, the clinical profile of patients with PE tends to differ from those with PR. Erosions have been observed more often in patients who do not fit the classic high-risk profile for coronary artery disease (CAD). These patients are frequently younger in age, often present with non-ST elevation ACS (NSTE-ACS), and have fewer traditional cardiovascular risk factors. It is now evident that improved risk-factor management with aggressive lipid-lowering therapies has reduced the incidence of large necrotic, rupture-prone plaques, thereby making erosion-related ACS relatively more frequent in the modern era. This review provides a comprehensive overview of PE—from epidemiology and pathophysiology to diagnosis and management—highlighting how OCT imaging has unveiled this entity and how it may guide personalized treatment strategies.

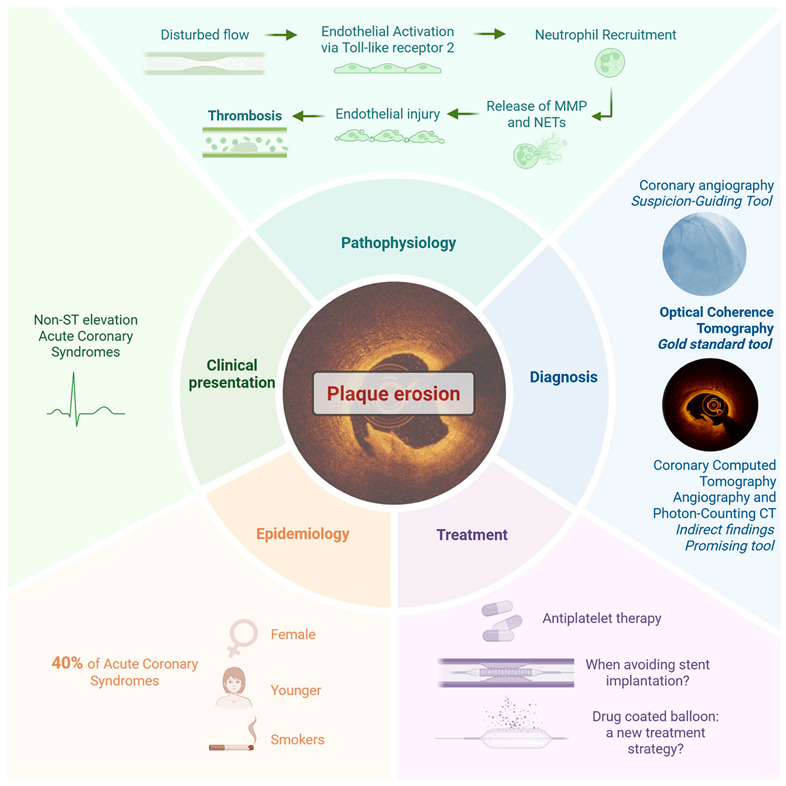



Central Illustration: summary of key aspects of plaque erosion, including its epidemiology, clinical presentation, pathophysiology, diagnosis, and treatment. Plaque erosion accounts for approximately 40% of ACS, especially in younger individuals, women, and smokers. Its pathophysiology involves endothelial injury without fibrous cap rupture, leading to platelet-rich thrombus formation. Diagnosis often relies on intracoronary imaging, with optical coherence tomography (OCT) as the gold standard. Treatment strategies may range from antiplatelet therapy alone to revascularization depending on clinical context.

## 2. Plaque Erosion: An Autopsy-Driven Shift in the Understanding of Acute Coronary Syndromes

The mechanisms underlying ACSs have been extensively investigated over the last several decades, with pathologists playing a central role in mapping the natural history of atherosclerotic disease. Among the most influential contributors in this field is Dr. Renu Virmani, whose meticulous autopsy-based research has been pivotal in defining the structural evolution of atherosclerotic plaques—from the earliest fatty streaks to the advanced lesions responsible for ACSs. Her work has been instrumental in identifying and characterizing three distinct mechanisms of plaque destabilization that can lead to acute thrombosis: PR, PE, and calcified nodules (CNs) [1].

Initially, PR was thought to be the dominant culprit in sudden coronary death and ACSs. However, through methodical histopathological studies of hearts from victims of sudden cardiac death, Virmani and colleagues redefined this landscape [1,2,3]. In a 1996 study, Farb et al. [4] revealed that PE—marked by thrombus formation over an intact fibrous cap and lacking a large lipid core—was a common yet overlooked cause of fatal coronary thrombosis. These lesions, rich in smooth muscle cells (VSMCs) and proteoglycans but sparse in macrophages, represented a distinct pathologic mechanism from classic PR. Building on this, Burke et al. [3] analyzed 51 women who died suddenly from coronary causes. They found a clear age- and risk-factor-dependent divergence in thrombotic mechanisms: PR predominated in postmenopausal women with hypercholesterolemia, while PE was more frequent in younger, often normolipidemic smokers and occurred in less obstructive lesions. Thrombi in PE cases were typically platelet-rich and non-occlusive, highlighting a less inflammatory and less stenotic process. Arbustini et al. [5] further corroborated these findings in a large autopsy cohort of acute MI patients, showing that PE accounted for 25% of cases, with a higher prevalence in women. These plaques maintained fibrous cap integrity and lacked a thrombus–core interaction. The conceptual breakthrough culminated in the comprehensive 2000 review by Virmani et al., “Lessons from Sudden Coronary Death”, which synthesized data from hundreds of autopsy cases. This work proposed a simplified morphological classification of atherosclerotic lesions, ranging from early intimal thickening to thin-cap fibroatheromas (TCFAs). Critically, it reaffirmed that only about one-third of fatal coronary thrombi were due to PR, while another third was caused by PE, and the remainder had no thrombus, suggesting alternative mechanisms of death such as lethal arrhythmia or ischemia from severe stenosis. The authors underscored that PE occurs in structurally intact, often fibrous plaques, and is likely triggered by superficial endothelial injury rather than deep inflammation and cap degradation. Together, these investigations illuminate the unique biology of PE—a mechanism defined by preserved fibrous cap integrity, minimal inflammation, high shear stress, and endothelial denudation. Clinically, this translates to a subset of ACS patients—often younger, female, and smokers—whose events are not caused by classic lipid-laden ruptured plaques. The recognition of PE as a discrete entity has since inspired the development of high-resolution intracoronary imaging and personalized treatment paradigms that aim to distinguish between erosion and rupture in vivo.

## 3. Epidemiology of Plaque Erosion: Prevalence and Patient Profiles

The epidemiological insights first suggested by autopsy-based investigations into PE have found solid confirmation in the era of intracoronary imaging. While early pathological studies had already drawn attention to PE as a significant and sex-based cause of coronary thrombosis, contemporary in vivo imaging—particularly through OCT—has not only validated those findings but also provided a more nuanced clinical and demographic portrait of this phenomenon.

A landmark study by Yamamoto et al. [6], which analyzed over 1200 ACS patients using pre-intervention OCT, established that PE accounts for approximately 40% of ACS cases, thereby affirming its role as a major contributor to ACSs. This prevalence mirrors the proportions observed in earlier post-mortem studies and definitively positions PE not as a marginal variant of PR but as a distinct pathological entity with its own clinical implications. One of the most consistent and striking observations across imaging studies is the greater prevalence of PE in women, particularly among younger age groups. In the extensive OCT-based cohort reported by Seegers et al. [7], the prevalence of PE was significantly higher in women < 50 years, while older women tended to exhibit morphological features more characteristic of PR, such as TCFAs and lipid-rich plaques. This age-dependent transition in plaque phenotype underscores the complex interplay between biological sex, hormonal status, and atherosclerotic progression. Complementing these observations, Araki et al. [8] provided further granularity by demonstrating that younger patients with PE typically exhibit fibrotic plaques rich in VSMC and proteoglycans, lacking significant lipid content or calcification. In contrast, older patients presenting with PE often showed more complex lesions, including lipid pools, cholesterol crystals (CCs), and CNs, and were more likely to have comorbidities such as diabetes, hypertension, and chronic kidney disease. These findings suggest the existence of at least two distinct phenotypes of PE: a proteoglycan-rich, “early-life” form and a lipid-rich, “late-life” form—each potentially requiring different therapeutic considerations. Further support for the age- and sex-specific patterns of PE comes from a multicenter study analyzing 1083 ACS patients with OCT-confirmed erosion or rupture. While the overall prevalence of erosion was similar between sexes, age-related differences were evident. Men ≤ 50 years and women aged 61–70 showed a significantly higher prevalence of PE, whereas both sexes experienced a marked decline beyond 70 years. Multivariate analysis confirmed that women <70 years had a significantly increased risk of PE, with men ≤50 years showing a similar trend [9]. These results reinforce that PE predominantly affects younger patients, particularly women before advanced age.

Across all studies, a recurring clinical signature emerges: patients with PE are more frequently smokers and less likely to exhibit traditional cardiovascular risk factors such as diabetes mellitus or dyslipidemia. This profile sharply contrasts with that of PR, which is typically associated with longstanding metabolic derangement and systemic inflammation. Notably, Yamamoto et al. [6] developed a predictive model incorporating clinical variables such as smoking status, preserved renal function, and higher hemoglobin levels to estimate the probability of PE in ACS patients, highlighting the feasibility of identifying erosion-prone individuals even before invasive imaging is performed.

Taken together, the findings from intravascular imaging (IVI) studies converge on a cohesive and clinically relevant narrative: PE is not only common but also demographically and biologically distinct. Its recognition challenges the longstanding dominance of the rupture-centric model of ACSs and opens the path toward personalized diagnostic and therapeutic strategies, informed by plaque morphology, patient profile, and the mechanism underlying ACSs.

## 4. Clinical Presentation of Plaque Erosion: How Does It Differ from Plaque Rupture?

PE is more typically associated with a clinical presentation of NSTE-ACS, rather than persistent ST-elevation MI (STEMI). Patients with PE erosion tend to exhibit preserved coronary flow, less myocardial injury, and white, platelet-rich thrombi, contributing to a generally milder clinical picture compared to PR [10]. However, recent studies have highlighted that the clinical expression of PE may vary meaningfully with age, likely reflecting both morphological and physiological differences in plaque behavior. In the large cohort studied by Yamamoto et al. [6], patients with PE more commonly presented with NSTE-ACS and exhibited lower levels of myocardial necrosis markers, indicative of smaller infarct size and more stable presentations. These findings are consistent with data from Jia et al. [11], who reported that 61.5% of erosion cases presented as NSTE-ACS compared to just 29.1% in PR. Similarly, Yonetsu et al. [12] observed that patients with intact fibrous caps—a hallmark of erosion—had lower peak myocardial necrosis marker levels and were more frequently diagnosed with NSTE-ACS, reinforcing the association between erosion and less severe myocardial injury. However, Araki et al. [8] revealed important age-dependent differences in the clinical presentation of PE that may at first appear counterintuitive. Their study showed that younger patients with PE typically exhibited fibrotic plaques, rich in VSMCs and proteoglycans, with minimal lipid content and calcification. Conversely, older patients with erosion often presented with more morphologically complex lesions, containing lipid pools, CCs, and CNs—features generally associated with higher thrombogenic potential. Despite this, younger individuals were more likely to present with STEMI, while older patients more commonly exhibited NSTE-ACS. This paradoxical age-related shift in clinical presentation was explained by two key considerations. First, older patients had significantly greater plaque burden, with the prevalence of diameter stenosis > 70% nearly doubling compared to younger individuals. This anatomical severity may lead to repeated subclinical ischemic episodes, which can trigger ischemic preconditioning, a phenomenon that renders the myocardium more resistant to acute ischemic injury, thereby reducing the likelihood of transmural AMI. Second, an additional hypothesis proposed by the present authors is that older patients are more frequently treated with chronic antiplatelet therapy, which may facilitate endogenous thrombolysis, preventing the formation of fully occlusive thrombi. These two mechanisms likely contribute to a blunted clinical expression of PE in elderly patients, shifting the presentation away from STEMI and toward NSTE-ACS, even when the underlying plaque morphology appears more complex and thrombogenic. This intriguing finding is further supported by Seegers et al. [7], which showed that younger women with PE were more likely to present with STEMI, while older patients—both male and female—showed a shift toward NSTE-ACS. Taken together, these studies suggest that while PE is classically linked to less severe clinical syndromes, its presentation is modulated by both biological age and therapeutic background. In younger patients—particularly women and smokers—acute coronary events more often present as STEMI, whereas in older individuals the combination of chronic ischemia, ischemic preconditioning, and ongoing antiplatelet therapy blunts symptom severity, leading to a milder presentation such as NSTE-ACS.

## 5. Pathophysiological Substrate of Plaque Erosion and Rupture: Two Distinct Mechanisms in ACS

For decades, the prevailing model of ACS has been built around the “vulnerable plaque” paradigm. In this model, ACS is classically driven by the rupture of TCFAs—plaques with large lipid cores, abundant macrophage infiltration, and thin fibrous caps, weakened by inflammatory degradation of interstitial collagen. These lesions, rich in tissue factor and inflammatory mediators, provoke massive thrombin generation upon rupture, leading to fibrin-rich occlusive thrombi and typically resulting in STEMI [13]. In contrast, PE represents a fundamentally different entity, both morphologically and immunologically. Rather than cap rupture, PE involves the loss or dysfunction of the endothelial layer overlying a proteoglycan- and VSMC-rich matrix, typically lacking lipid cores and macrophage infiltration. The resulting thrombus is often platelet-rich (“white thrombus”) and non-occlusive, more frequently leading to NSTE-ACS [13]. The underlying mechanisms that lead to superficial erosion remain incompletely understood but are increasingly being elucidated. A central hypothesis posits that the process begins with endothelial injury or dysfunction. In contrast to PR, which is driven largely by chronic inflammation mediated by adaptive immune cells and cytokines that weaken the fibrous cap, erosion may stem from innate immune activation coupled with hemodynamic stress. One proposed initiator is Toll-like receptor 2 (TLR2), an innate immune sensor that is upregulated in endothelial cells in regions of disturbed flow. Experimental stimulation of TLR2 with its ligands induces endothelial expression of adhesion molecules acting as potent neutrophil chemoattractants. This activation is milder than that induced by classical pro-inflammatory cytokines like TNF-α but is sufficient to create a low-grade, smoldering inflammatory state [13,14]. As this endothelial activation progresses, it can lead to desquamation of the endothelial layer. This loss of endothelial integrity is potentiated by decreased expression and functional disruption of junctional proteins which maintain endothelial barrier function. Compounding this injury, neutrophils are recruited to the site of endothelial disruption and release neutrophil extracellular traps (NETs) through a specialized form of cell death known as NETosis [13]. NETs, composed of DNA strands decorated with cytotoxic and prothrombotic proteins such as myeloperoxidase (MPO), neutrophil elastase, and cathepsin G, serve multiple pathological roles. They promote local thrombosis by trapping platelets, activating the coagulation cascade, and amplifying inflammation. NETs can also incorporate tissue factor from the bloodstream, enhance IL-1α activation, and degrade basement membrane components, exacerbating endothelial injury [13].

This pathophysiological distinct substrate was further clarified by the OPTICO-ACS studies, which provided detailed insights into the distinct inflammatory mechanisms and clinical outcomes associated with plaque rupture (rupture fibrous cap, RFC) and plaque erosion (intact fibrous cap, IFC) in ACSs [15,16,17]. The OCT study conducted by Leistner et al. [15] demonstrated that RFC-ACS lesions are characterized by higher lipid content, thinner fibrous caps, and extensive systemic inflammation, with elevated levels of interleukin-6 (IL-6) and interleukin-1β. In contrast, IFC-ACS lesions have a thicker fibrous cap, lower lipid content, and are often located near coronary bifurcations. IFC-ACS involves local immune activation with increased CD4+ and CD8+ T cells and elevated cytotoxic molecules such as granzyme A, perforin, and granulysin. Moreover, experimental models demonstrated increased adhesion of CD8+ T cells under disturbed flow conditions, mimicking coronary bifurcations. This study emphasizes the presence of distinct immunological signatures between IFC-ACS and RFC-ACS, highlighting that PE may be driven by endothelial damage caused by cytotoxic T cells, a process further exacerbated by turbulent flow conditions. Another study, conducted by Meteva et al., delves deeper into the underlying mechanisms of IFC-ACS [16]. The research identified TLR 2-mediated neutrophil activation as a pivotal driver of endothelial damage in IFC-ACS. In these lesions, neutrophils exhibit heightened TLR2 expression, stimulated by elevated levels of hyaluronic acid and disturbed flow conditions near bifurcations. Activated neutrophils release matrix metalloproteinase 9 (MMP9), which intensifies endothelial detachment and cell death, ultimately promoting thrombus formation [18]. Finally, a recently published investigation compared clinical outcomes between RFC-ACS and IFC-ACS and found that patients with RFC-ACS have a higher residual inflammatory risk and worse outcomes, with a two-year major adverse cardiac events (MACEs) rate of 26.7%, compared to 14.3% in IFC-ACS. The inflammatory burden in RFC-ACS was significantly higher, driven by systemic activation of innate immune pathways, including the IL-1β to IL-6 axis. In contrast, IFC-ACS patients exhibited a lower inflammatory profile and localized immune responses [17]. In conclusion, the OPTICO-ACS studies collectively reveal that RFC and IFC in ACS are underpinned by distinct pathophysiological mechanisms and inflammatory responses. While RFC-ACS involves intense systemic inflammation and a high-risk profile, IFC-ACS features localized immune activation with a lower overall inflammatory burden. These findings underline the importance of lesion-specific and patient-specific approaches to therapy, emphasizing personalized treatment strategies for optimizing outcomes in ACS patients.

Additionally, shear stress plays a mechanical role. Computational fluid dynamics studies indicate that regions of high shear stress adjacent to zones of low and oscillatory shear are hotspots for thrombus formation in eroded plaques. This suggests that hemodynamic factors interact with molecular signals to localize injury and thrombosis. Another relevant mechanism is endothelial-to-mesenchymal transition (EndMT), a process by which endothelial cells acquire a mesenchymal phenotype, lose polarity and adhesion, and invade the intimal space. This phenotypic shift may further impair endothelial function and promote matrix remodeling, setting the stage for thrombus formation.

Supporting this evidence, combining computational fluid dynamics and the OCT study by Yamamoto et al. [19] showed that thrombus formation in PE was consistently localized between regions of high endothelial shear stress at the minimal lumen area and zones of high oscillatory shear index downstream. This flow pattern, present in 78% of PE cases studied, reflects a biomechanical environment that fosters platelet activation at peak shear, followed by thrombus propagation in regions of disturbed, recirculating flow. Chronic low shear stress is known to induce endothelial inflammation and apoptosis, while supraphysiological ESS can lead to mechanical injury and endothelial denudation [20].

These mechanical forces act synergistically with immune signals, such as TLR2 activation, to promote endothelial dysfunction and trigger the release of NETs, web-like DNA structures that propagate thrombosis by carrying proteolytic enzymes, oxidants, and tissue factor [21]. Finally, these findings support a “two-hit” model of superficial erosion: first, a low-grade activation of endothelial cells via innate immune stimuli and mechanical forces; second, the recruitment of neutrophils and formation of NETs, leading to endothelial detachment and thrombus formation [13].

In summary, PR and PE represent two pathophysiologically distinct pathways to coronary thrombosis. While PR is driven by lipid accumulation, macrophage-driven inflammation, and fibrous cap degradation, PE involves mechanical and immune-mediated endothelial injury, localized at sites of altered flow dynamics. Recognizing these differences not only advances our understanding of ACS but also paves the way for precision medicine, allowing clinicians to align treatment strategies with lesion-specific biology.

## 6. Diagnosis of Plaque Erosion: The Role of Invasive and Non-Invasive Imaging

Identifying PE in patients with ACS is clinically crucial, as this mechanism differs substantially in pathophysiology and treatment response from PR. While coronary angiography remains the primary imaging modality for assessing coronary anatomy and guiding revascularization, it lacks the resolution to directly visualize the morphological features that differentiate erosion from rupture. However, when integrated with the clinical context and patient characteristics, angiography may offer valuable indirect indicators that raise suspicion for plaque erosion and prompt further investigation. Specific angiographical features may suggest PE, particularly in younger patients, smokers, or those without extensive CAD. In such cases, angiography supported by quantitative coronary angiography (QCA) can offer additional insight, helping to identify patients in whom IVI techniques, such as OCT, should be selectively employed. The following section will examine the respective roles of currently available imaging techniques, including coronary angiography, OCT, intravascular ultrasound (IVUS), near-infrared spectroscopy (NIRS), and coronary computed tomography angiography (CCTA), in the diagnosis of PE and their contribution to mechanism-guided management of ACSs.

### 6.1. Plaque Erosion and Coronary Angiography: A Non-Diagnostic but Suspicion-Guiding Tool

Coronary angiography remains a cornerstone in the evaluation and management of ACSs, yet it has well-recognized limitations in discerning the underlying mechanisms of plaque disruption, namely distinguishing between PR, PE, and CNs. These distinctions are clinically relevant, as they carry important therapeutic implications. Nevertheless, emerging evidence suggests that certain angiographic features, particularly when integrated with clinical presentation and patient characteristics, may offer valuable clues for suspecting PE. In such cases, the selective use of IVI modalities, such as OCT, can provide critical insights into the morphological substrate of the culprit lesion and support more personalized, mechanism-guided treatment strategies.

In a large multicenter analysis of patients presenting with non-ST-elevation ACS, those with OCT-confirmed PE demonstrated angiographic profiles that were notably less complex than those with PR. Erosions were associated with lower angiographic risk scores, including Jeopardy, Gensini, and Syntax scores, and were more frequently located in the mid-left anterior descending (LAD) artery. These lesions exhibited less frequent B2/C lesion morphology and reduced prevalence of an angiographically visible thrombus or calcification. Using QCA, the authors identified that erosions were associated with a smaller reference vessel diameter and shorter lesion length [22]. Complementary findings were reported in a focused angiography-OCT study of STEMI patients, which highlighted the geometric characteristics of erosion-prone plaques. This study compared 484 culprit PE lesions with 1132 non-culprit plaques and demonstrated that culprit erosions were associated with significantly higher diameter stenosis and longer lesion length. Notably, QCA measurements also showed smaller minimal lumen diameters in culprit erosions [23]. Moreover, these angiographic findings were spatially contextualized: most culprit PEs were clustered within 40 mm of the LAD ostium and frequently occurred proximal to a bifurcation, indicating a geometrical predilection for specific coronary segments. Importantly, minimal lumen area (MLA) < 2.51 mm^2^ and area stenosis (AS) >64.02%—measured using OCT but suggested by QCA geometry—were identified as independent predictors of culprit PE [24].

Taken together, these studies suggest a refined role for coronary angiography, not as a definitive diagnostic tool for PE but as a clinical compass that can orient suspicion. When specific angiographic and QCA-derived features are observed, such as focal, high-grade stenosis in the mid-LAD, simple lesion morphology, absence of calcification or a large thrombus, and lower global angiographic scores, they should prompt the clinician to consider plaque erosion in the differential diagnosis. In this regard, QCA emerges as an underutilized but highly accessible adjunct that adds quantitative depth to the qualitative impressions formed during angiographic interpretation. Ultimately, while only IVI can definitively characterize plaque morphology, the ability to suspect PE based on angiographic and QCA findings allows for more judicious, mechanism-targeted use of technologies like optical coherence tomography.

### 6.2. Assessing Plaque Erosion Using Optical Coherence Tomography: A Diagnostic Game Changer

**Figure 1:**
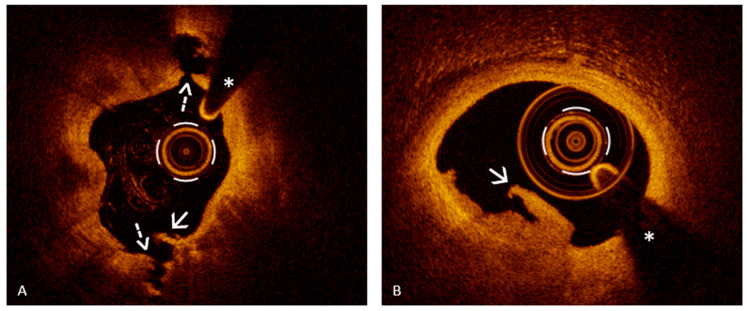
Plaque rupture and plaque erosion. Panel (**A**): Plaque rupture with evidence of fibrous cap discontinuity resulting in two distinct cavities in the vessel wall at 12 and 6 o’clock (*dashed arrow*), with irregularities of the luminal surface (solid arrow). The underlying plaque is lipid-rich, characterized by a low-backscattering zone with poorly defined borders (extending from 12 to 6 o’clock) and a high-backscattering fibrous cap of variable thickness. Panel (**B**): Plaque erosion with a mixed thrombus overlying an intact fibrous cap. *The asterisks indicate the guide wire artifact. All images come from the authors’ personal archive*.

In the modern landscape of ACS management, OCT has emerged as an indispensable imaging modality that surpasses traditional coronary angiography in diagnostic precision. While angiography remains the frontline tool for identifying obstructive lesions, its limitations in characterizing plaque morphology and detecting the underlying mechanisms of plaque destabilization have become increasingly evident—particularly in patients with ambiguous angiographic findings or non-obstructive culprit lesions [10]. Particularly, the advent of OCT has revolutionized the diagnosis of PE in vivo. The diagnostic goal of this instrumental imaging tool is to determine whether the culprit lesion has a visible cap rupture or not, as this is the key distinction between PR and PE. The hallmark OCT finding for PE is the presence of a luminal thrombus, appearing as irregular masses protruding into the lumen, attached to an intact plaque surface, without any evidence of fibrous cap rupture [25,26]. By contrast, an OCT-confirmed PR is identified when a break in the fibrous cap is clearly visualized, usually with a cavity or deep gap connecting to a lipid core (Figure 1, Panel A) [25,26]. Finally, if OCT shows a thrombus without fibrous cap rupture at the culprit site in an ACS patient, the lesion can be classified as an OCT-defined PE (Figure 1, Panel B) [26]. Moreover, two distinct types of OCT-defined erosion have been described: definite and probable erosion. Definite erosion is diagnosed when a luminal thrombus is clearly visualized over an intact fibrous cap that covers an atherosclerotic plaque, typically containing lipid or calcium [25]. In contrast, probable erosion refers to the presence of a thrombus or irregularities on the luminal surface in segments of the vessel that appear otherwise normal, without clear evidence of underlying plaque [25].

**Figure 2:**
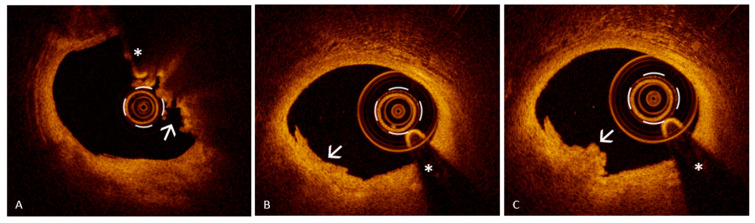
Types of thrombi in plaque erosion. Panel (**A**): Plaque erosion over a likely lipid-rich plaque, associated with a red thrombus (white arrow), characterized by high backscattering and significant light attenuation, resulting in shadowing that obscures the underlying plaque. Panel (**B**): Erosion with a white thrombus (white arrow), showing more moderate signal intensity and less attenuation, allowing visualization of the underlying lipid plaque. Panel (**C**): Erosion with a mixed thrombus (white arrow), which displays intermediate characteristics, combining features of both red and white components. *The asterisks indicate the guide wire artifact. All images come from the authors’ personal archive*.

Since the diagnosis of definite erosion relies on the visualization of thrombi overlying an intact fibrous cap, recognizing them and distinguishing their features becomes a fundamental aspect of OCT image interpretation. In this scenario, OCT plays a key role, offering high-resolution visualization that surpasses other imaging modalities. It is considered the reference standard for identifying thrombotic material within the coronary arteries, which may appear as masses adherent to the vessel wall or as mobile structures within the lumen [25,26,27]. Importantly, OCT enables the differentiation between a red thrombus, which is predominantly composed of erythrocytes, and a white thrombus, which is rich in platelets, based on their distinct optical signatures. Red thrombi typically produce strong signal reflection (high backscatter) and significant light attenuation, often leading to shadowing beyond the thrombus (Figure 2, Panel A). In contrast, white thrombi exhibit a more moderate signal intensity with less attenuation, allowing better visualization of underlying structures (Figure 2, Panel B). A third category, the mixed thrombus, displays intermediate characteristics, combining features of both red and white components (Figure 2, Panel C) [25]. Notably, white thrombi, composed primarily of platelets, are more commonly associated with PE, whereas red thrombi, rich in erythrocytes, are more frequently observed in cases of PR, reflecting their distinct underlying pathophysiology [28]. However, thrombus composition does not contribute to the diagnostic differentiation between plaque rupture and erosion with OCT and should therefore be interpreted as a supportive but non-discriminatory finding.

Moreover, it is essential to recognize that the presence of a thrombus can be underestimated during OCT imaging, particularly in patients with ACS who have received thrombolytic agents or potent antithrombotic therapy, as partial or complete thrombus resolution may occur prior to imaging. Furthermore, thrombus visualization is a critical criterion for confirming certain plaque phenotypes: it is required to establish a diagnosis of definite OCT erosion, whereas its presence is not necessary to classify a lesion as probable OCT erosion. This highlights the importance of careful image interpretation and timing of OCT acquisition relative to clinical presentation and pharmacologic treatment.

It is also important to note that diagnosing PE with OCT requires more than simply ruling out fibrous cap rupture. A rare yet clinically significant lesion that may mimic erosion is the eruptive CN, a distinct pathological substrate associated with intracoronary thrombosis. Although relatively uncommon, eruptive CNs account for approximately 2–7% of culprit lesions in ACSs [11], and their appearance on OCT can closely resemble that of PE, particularly when cap rupture is not clearly evident. CNs are characterized by nodular calcium protruding into the vessel lumen and can be classified into non-eruptive and eruptive forms based on their OCT features [26,27]. Non-eruptive CNs are typically covered by a thick fibrous cap and lack a thrombus, while eruptive CNs display features of fibrous cap disruption, superficial calcium protrusion, and an overlying thrombus. On OCT imaging, eruptive CNs present as irregular, protruding calcific masses with an associated thrombus and significant signal attenuation [25,26,27]. These characteristics can simulate the appearance of a thrombus overlying an intact plaque, potentially leading to misdiagnosis as PE. OCT plays a pivotal role in distinguishing these entities by providing high-resolution, cross-sectional visualization of the entire calcified segment. Notably, CNs tend to arise within heavily calcified plaques [25]; thus, accurate diagnosis also benefits from considering the overall plaque phenotype observed along the entire OCT pullback. Evaluating the broader atherosclerotic context, including the presence of extensive calcification proximal and distal to the lesion, can strengthen the suspicion of an eruptive CN rather than PE. While in many cases the presence of protruding nodular calcium into the lumen makes the diagnosis straightforward, this protrusion is not always clearly defined: when it is mild or irregular, it may be more difficult to differentiate from a thrombus on an otherwise intact plaque, increasing the risk of misclassification. Therefore, a thorough and contextual interpretation of OCT images, beyond the focal lesion, is essential to accurately identify eruptive CNs and avoid confusion with PE, ensuring appropriate therapeutic decisions are made.

**Figure 3:**
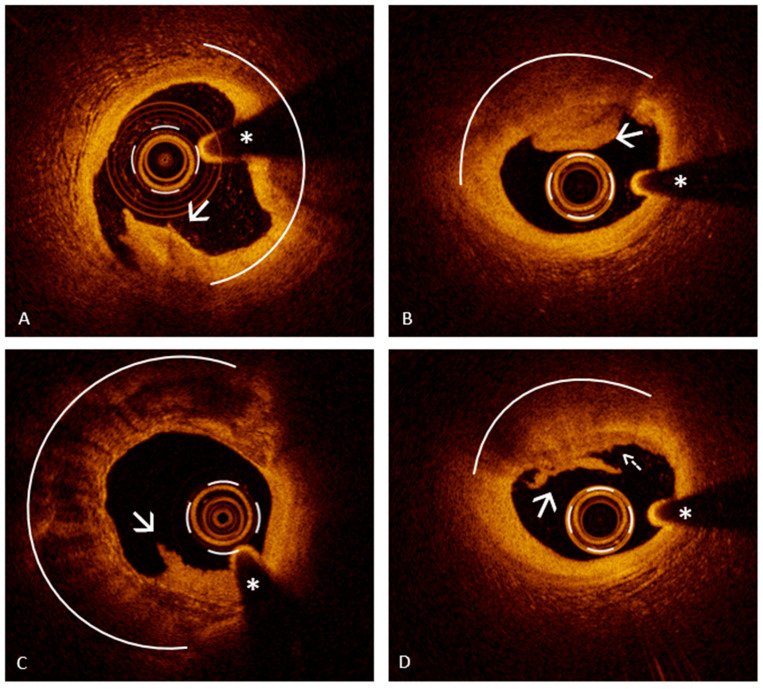
Plaque phenotype underlying plaque erosion. Panel (**A**): PE with a mixed thrombus (white arrow) over a fibrous plaque, characterized by loss of the typical three-layered architecture and appearance of a homogeneous high-intensity signal region (white curved line). Panel (**B**): PE with a mixed thrombus (white arrow) that allows visualization of the underlying lipid plaque, identified as a low-backscattering zone with poorly defined borders and an angular extension < 90° (white curved line). Panel (**C**): PE with a white thrombus in the setting of a stenosis with diffuse calcification. Calcium appears as a region with low backscattering, heterogeneous signal, and sharply defined borders. The calcified arc exceeds 90° (white curved line), consistent with diffuse calcification. Panel (**D**): PE with a mixed thrombus (solid white arrow) over a lipid plaque (white curved line), which shows features of healing (dashed arrow), such as a distinct superficial layer with heterogeneous optical signal and clear demarcation from the underlying tissue. *The asterisks indicate the guide wire artifact. All images come from the authors’ personal archive*.

OCT provides not only the means to identify PE but also the ability to contextualize it within the broader framework of the patient’s atherosclerotic disease. Typically, plaques associated with erosion show a relatively preserved lumen and a lower plaque burden when compared to those involved in PR. Another key distinguishing feature is the pattern of vascular remodeling: erosive lesions are more often associated with negative remodeling, whereas positive remodeling is more frequently observed in rupture-prone plaques [11]. Finally, beyond PE detection, OCT offers critical insight into the plaque phenotype underlying erosion. While the substrate for PR is well defined, most often corresponding to TCFA, the setting for PE is less predictable, as it can arise from a range of plaque types not typically associated with vulnerability features (Figure 3) [4,28,29].

In conclusion, OCT has become an essential tool for the in vivo identification of PE, offering high-resolution imaging that allows clinicians to distinguish it from other mechanisms of ACS. It enables precise detection of a thrombus overlying an intact fibrous cap, a hallmark of erosion, and helps differentiate it from PR or mimicking entities such as eruptive CNs. OCT also allows for thrombus characterization and provides insight into the underlying plaque phenotype. By integrating morphological details with lesion context, OCT supports a more accurate, mechanism-based diagnosis, guiding personalized therapeutic decisions in patients with ACS.

### 6.3. Diagnostic Strategies with Intravascular Ultrasound and Near-Infrared Spectroscopy

While OCT is the gold standard for in vivo erosion diagnosis, other modalities are being explored. High-definition IVUS (HD-IVUS) can sometimes hint at erosion by showing a plaque with no clear rupture and only mild surface irregularities or a layered thrombus and a pattern of negative remodeling. Novel techniques like combined NIRS-IVUS are also under research to better characterize plaque composition.

Conventional IVUS lacks the spatial resolution necessary to reliably identify the subtle features that characterize PE, such as an intact fibrous cap and superficial thrombus. However, its high-definition variant (HD-IVUS) offers improved tissue penetration and can be useful in scenarios where OCT is limited, such as in patients with renal dysfunction, large vessel diameters, or suboptimal blood clearance [30]. Despite its lower spatial resolution (100–150 µm) compared to OCT, HD-IVUS has shown some potential in detecting features suggestive of erosion. Cuesta et al. [31] explored the potential of this IVI modality in a series of patients with OCT-confirmed erosion. HD-IVUS was able to identify minor surface irregularities and a superficial thrombus in the absence of signs of rupture, suggesting some potential utility when OCT is unavailable. Nevertheless, due to its limited ability to differentiate between plaque components and thrombus layers, HD-IVUS remains a supplementary tool rather than a primary diagnostic modality. Similarly, Higuma et al. [28] demonstrated the complementary value of combining OCT and IVUS in the assessment of culprit lesions during primary PCI in AMI patients. While OCT remains the modality of choice for evaluating fibrous cap integrity and thrombus presence—crucial for distinguishing PE from PR—IVUS contributes by assessing overall plaque burden and vessel remodeling. As previously described, PE is often associated with lower plaque burden and negative remodeling, both of which are readily evaluated by IVUS.

The integration of virtual histology with IVUS (VH-IVUS) further refines plaque characterization. In cases of PE, the underlying plaques are predominantly fibrotic, often associated with a white thrombus, and tend to have an eccentric distribution. Conversely, PR is generally linked to lipid-rich plaques, a red thrombus, and a more concentric morphology [30]. Therefore, although IVUS lacks the resolution of OCT, it can still provide valuable information regarding the extent of atherosclerotic disease, patterns of vessel remodeling, and the plaque phenotype—factors that may indirectly support the identification of PE. In its high-definition form, IVUS may also detect luminal surface irregularities, albeit with significantly lower precision than OCT. In conclusion, IVUS can be considered a useful alternative when OCT is contraindicated or unavailable, but it should not be regarded as the imaging modality of choice for diagnosing PE.

NIRS is a catheter-based technique that assesses the chemical composition of the vessel wall by detecting the lipid core burden index (LCBI) [32]. When combined with IVUS (as in NIRS-IVUS), it provides both compositional and morphologic information. An intriguing study by Terada et al. [33] evaluated the utility of NIRS-IVUS in differentiating between PR, PE, and CNs in patients with AMI. Using OCT as the reference standard, the authors analyzed 244 patients and found that PE could be identified based on a set of indirect criteria rather than a single definitive feature. Specifically, erosion was characterized by the absence of an intraplaque cavity (a hallmark of PR), the absence of convex calcification (typical of CN), and the presence of a visible plaque with luminal irregularities or a superficial thrombus. Importantly, lesions classified as erosion also demonstrated a lower lipid content on NIRS. By combining these morphological and compositional parameters, the authors developed a diagnostic algorithm that showed excellent performance in identifying PE, with high sensitivity and specificity. These results suggest that while OCT remains the gold standard for detecting PE, particularly due to its ability to directly visualize fibrous cap integrity, NIRS-IVUS can offer a reliable alternative in settings where OCT is not feasible. The combination of negative findings on IVUS with a low lipid burden on NIRS can support the diagnosis of PE and guide clinical decision making in ACS.

The ability of NIRS to quantify lipid content within coronary plaques has provided a valuable addition to our understanding of PE. Much like previous insights gained from OCT-based studies, NIRS data have revealed that not all PEs arise from fibrous, lipid-poor substrates as traditionally believed. In fact, a subset of OCT-defined erosions demonstrates significant lipid presence on NIRS, indicating that erosion can also originate from lipid-rich plaques. This emerging evidence underscores the heterogeneity of erosion as a pathological process [30]. Clinically, this variability may have important implications. Lipid-poor erosions could represent a more stable phenotype, potentially responsive to conservative medical management. Conversely, lipid-rich erosions may carry a higher risk for progression or recurrent events, warranting more aggressive monitoring or intervention. In this context, NIRS offers a practical advantage, not only by refining the biological characterization of the lesion but also by aiding in the stratification of erosion subtypes in settings where OCT is either unavailable or contraindicated. As part of a multimodal imaging strategy, NIRS contributes critical compositional information that supports more personalized treatment approaches in complex ACS cases.

### 6.4. Diagnostic Strategies with Coronary Computed Tomography Angiography

CCTA has emerged as a cornerstone in the non-invasive assessment of CAD, offering a direct anatomical evaluation of both the coronary lumen and vessel wall through the intravenous administration of a contrast agent. Over the past two decades, technological advancements in computed tomography (CT) scanners, coupled with improved image acquisition and post-processing techniques, have significantly enhanced the accuracy and utility of CCTA, offering insights into coronary anatomy, plaque phenotype, functional significance of lesions, and vascular inflammation. This expanded role goes beyond simply detecting luminal stenosis, allowing clinicians to perform more accurate risk stratification and personalized treatment planning [34,35]. Although CCTA is primarily recommended in the evaluation of patients with CCS [36], current European Society of Cardiology (ESC) Guidelines expanded its role to include selected ACS settings. Specifically, in patients presenting with chest pain but inconclusive troponin levels, no ECG changes, and no recurrent symptoms, the incorporation of CCTA as part of the initial diagnostic workup should be considered (Class IIa, A) [37]. In this context, CCTA is not merely a tool for detecting obstructive CAD but also takes on the challenge of identifying the underlying mechanisms of ACS.

In the contemporary era of cardiovascular medicine, CCTA has evolved well beyond its original role as a purely anatomical tool for assessing stenosis severity. Thanks to continuous technological advances and refined image analysis techniques, CCTA now plays a central role in characterizing coronary plaque phenotype, identifying features of vulnerability, and even capturing CAD biological activity [38]. CCTA enables non-invasive visualization of plaque morphology, allowing the detection of high-risk characteristics such as low-attenuation plaque (a surrogate for a lipid-rich necrotic core), positive remodeling (indicative of compensatory vessel enlargement), spotty calcifications, and the so-called napkin-ring sign, a ring-like low attenuation halo that often signals underlying necrotic content. These imaging markers, when present, are indicative of heightened plaque vulnerability and an elevated risk of future acute coronary events. This association was notably confirmed in a post hoc analysis of the SCOT-HEART trial [39], where the identification of high-risk plaque features on CCTA, including low-attenuation plaque, positive remodeling, spotty calcifications, and the napkin-ring sign, was significantly correlated with an increased incidence of subsequent cardiovascular events. Importantly, these prognostic associations held true even in individuals without flow-limiting stenoses, emphasizing the value of advanced plaque characterization in risk stratification beyond conventional anatomical assessment. A major technological leap in this domain has been the ability of CCTA to evaluate pericoronary adipose tissue (PVAT), not merely as a passive structural component but as a dynamic, metabolically active tissue that reflects and influences coronary inflammation. Through radiomic analysis, CCTA can assess PVAT both qualitatively and quantitatively, shedding light on the bi-directional crosstalk between inflamed vascular walls and surrounding fat. In this context, the fat attenuation index (FAI) has emerged as a novel, CT-derived biomarker that captures spatial changes in PVAT attenuation, thereby providing a non-invasive window into vascular inflammation [40]. This novel imaging biomarker offers a unique, non-invasive means of evaluating vascular inflammation, a process that plays a central role in plaque destabilization. Further strengthening this paradigm, the CRISP-CT study [41], which included over 3,900 patients, demonstrated that elevated perivascular FAI was a strong and independent predictor of both cardiac mortality and MACE. Notably, this association remained significant regardless of traditional cardiovascular risk factors or the extent of plaque burden, highlighting the incremental prognostic value of FAI. These findings position FAI not merely as a structural or anatomical marker but as a functional indicator of active disease, capable of enhancing current risk stratification models and supporting a more biologically informed approach to cardiovascular prevention and therapy.

CCTA has clearly established itself as a valuable tool for assessing the anatomical severity of coronary stenoses, as well as for differentiating between plaque phenotypes, detecting high-risk features of vulnerability, and even indirectly evaluating coronary inflammation through radiomic analysis of PVAT. However, a critical question remains: can CCTA identify the specific mechanisms underlying ACS and, in particular, PE? While CCTA excels in structural characterization and offers important functional insights, its ability to distinguish among the pathophysiological substrates of ACS remains limited. Unlike intravascular imaging modalities such as OCT, which can directly visualize fibrous cap integrity and thrombus morphology, CCTA lacks the spatial resolution necessary to confirm these fine structural details. Nonetheless, ongoing research is exploring how certain indirect features on CCTA, such as the absence of high-risk plaque characteristics or a preserved vessel architecture in the context of ACS, may contribute to raising clinical suspicion of plaque erosion in selected scenarios.

Recent investigations have explored the potential of CCTA to differentiate PE from PR. In a study by Suzuki et al. [42], patients with OCT-confirmed PE exhibited significantly lower total plaque volumes and a reduced prevalence of high-risk plaque features, such as positive remodeling, low-attenuation plaque, spotty calcification, and the napkin-ring sign, compared to those with PR. Notably, a total plaque volume less than 116 mm^3^ and the presence of one or no high-risk features emerged as independent predictors of PE. These findings were supported by the combined OCT-CCTA study by Niida [43], which examined whether CCTA could help distinguish PE from PR in patients with NSTE-ACS by assessing overall coronary plaque burden. In a cohort of 232 patients who underwent both CCTA and OCT before PCI, the authors found that patients with PR had a significantly higher total atherosclerotic burden than those with PE. This included greater volumes of non-calcified, low-density non-calcified, and calcified plaques, both at the culprit lesion and throughout the coronary tree. These results suggest that PE is typically associated with a lower and less lipid-rich plaque burden, while PR reflects more extensive and vulnerable atherosclerosis.

Together, these studies suggest that CCTA may enable an indirect diagnosis of PE, primarily through the quantification of overall plaque burden, which appears consistently lower in erosion than in rupture, and through characterization of the lesion phenotype. Erosive plaques tend to lack extensive lipid-rich necrotic cores and other hallmarks of vulnerability, presenting instead with a more fibrous, less inflamed profile. While CCTA cannot directly visualize fibrous cap integrity or thrombi, its ability to assess these morphological and compositional differences may help raise suspicion for plaque erosion in appropriate clinical contexts. Finally, in the context of multimodality imaging and artificial intelligence integration, CCTA may become a valuable tool in pre-selecting patients for invasive OCT, especially when lesion morphology and plaque burden suggest a non-rupture etiology.

Among the most advanced applications of CCTA, the assessment of PVAT through the FAI—a radiomic marker of local vascular inflammation—has opened new avenues for understanding plaque biology. Since inflammation contributes differently to rupture and erosion, FAI may enhance the diagnostic precision of CCTA when differentiating these two ACS mechanisms. In this context, Nagamine et al. [44] explored whether combining CCTA-derived plaque characteristics with FAI could aid in the non-invasive identification of PE. This study enrolled 186 patients with NSTE-ACS who underwent CCTA prior to invasive coronary angiography and OCT. The authors aimed to determine whether CCTA-derived morphological and inflammatory features could reliably identify OCT-defined PE, specifically characterized by the presence of an IFC. Their analysis showed that lesions with IFC had a significantly lower prevalence of high-risk plaque features, such as low-attenuation plaque, positive remodeling, napkin-ring sign, and spotty calcification, all of which are commonly associated with PR. Furthermore, IFC lesions were strongly associated with a zero coronary artery calcium score and with lower PVAT inflammation, quantified using FAI. These characteristics suggest a less inflamed and more stable atherosclerotic profile in patients with PE compared to those with rupture. Importantly, Nagamine et al. proposed a simple predictive scoring system that combined the absence of high-risk plaque features, low FAI, and zero calcium score. This model demonstrated good discriminatory ability in identifying patients with PE, offering a potential non-invasive method to triage patients and to guide decisions about early invasive management or conservative therapy. These findings support the growing role of multi-parametric CCTA as a valuable complement to IVI, particularly in settings where OCT is not immediately available.

Following this growing body of evidence supporting the role of multi-parametric CCTA in the indirect identification of PE, it is reasonable to speculate that photon-counting computed tomography (PCCT) could represent the next technological leap capable of overcoming several intrinsic limitations of conventional CCTA. A major advancement in CT imaging, PCCT utilizes photon-counting detectors that measure individual X-ray photons and their respective energy levels, in contrast to traditional energy-integrating detectors. This approach confers multiple benefits: significantly enhanced spatial resolution, improved contrast-to-noise ratio, reduced image noise, and the capability for intrinsic spectral imaging—all while potentially lowering radiation exposure. These features allow for better tissue differentiation, with a marked reduction in common artifacts such as blooming from calcifications or stents. Spectral data acquisition within a single scan further enables more accurate differentiation between lipid-rich, fibrous, and calcified tissue components based on their unique energy-dependent attenuation profiles, offering a more refined assessment of plaque composition and total atherosclerotic burden [45,46]. In this context, PCCT emerges not only as an advanced anatomical imaging modality but also as a non-invasive tool for high-fidelity plaque phenotyping, with the potential to identify subtle morphological features, such as positive remodeling or low-attenuation areas, more accurately than current CCTA techniques. These traits are critical in recognizing high-risk plaques that may be predisposed to rupture or erosion. Importantly, the ultra-high spatial resolution of PCCT could provide greater insight into the pathophysiological mechanisms underlying ACS, including PE. While PCCT still lacks the microscopic resolution of intravascular imaging techniques like OCT, its ability to detect minute differences in tissue characteristics and to assess vascular inflammation through spectral analysis may enhance the identification of plaques with a more fibrous and stable profile—typical of erosion—versus the lipid-rich, inflamed phenotypes associated with rupture.

Despite these promising advantages, the clinical use of PCCT in the ACS setting still faces important limitations. In unstable patients or those with high-risk clinical features, the time-sensitive nature of diagnosis and the potential need for immediate intervention may favor invasive strategies. However, in selected low- to intermediate-risk patients with atypical presentations or inconclusive biomarkers, PCCT could play a valuable role in the early, non-invasive stratification of ACS mechanisms. Its capacity to integrate morphological, compositional, and potentially inflammatory information from a single PCCT scan position is a future cornerstone of precision cardiovascular imaging.

This potential has been vividly illustrated in the first reported clinical case describing the use of PCCT in a patient with STEMI [47]. In this case, PCCT was successfully employed to simultaneously assess coronary anatomy, perivascular inflammation through FAI, and myocardial injury via iodine-based extracellular volume mapping. The scan not only identified the culprit artery and its inflammatory status but also revealed the extent and nature of myocardial damage, including the presence of microvascular obstruction and the transmurality of infarction. Pending broader validation, this approach may help guide revascularization strategies and tailor long-term management based on the biological profile of both plaque and myocardium. Although the underlying pathophysiological mechanism of ACS—specifically whether PR or PE—was not determined in this case, it nevertheless offers a compelling proof of concept: PCCT is technically feasible and clinically informative in selected cases of ACS. Its capacity to integrate detailed anatomical, inflammatory, and myocardial assessments non-invasively opens new possibilities for diagnostic and prognostic refinement. In the future, if tested in larger cohorts of patients with ACS and validated against IVI modalities such as OCT, PCCT could potentially emerge as a useful tool for identifying the underlying mechanism of acute events, including PE. Such developments would mark a significant step forward in non-invasive precision medicine approaches to ACS, but future studies are essential to confirm its diagnostic performance and to establish standardized criteria for differentiating ACS mechanisms based on PCCT imaging biomarkers.

In summary, OCT remains the gold standard for diagnosing PE, offering direct visualization of the plaque–thrombus interface and fibrous cap status. IVUS and NIRS provide valuable complementary information on plaque structure and composition, especially when OCT is not feasible. Meanwhile, CCTA shows potential as a non-invasive screening tool, particularly when augmented by quantitative plaque analysis and FAI. As imaging technologies evolve, the integration of multiple modalities may enable earlier, safer, and more accurate identification of PE, paving the way for more personalized and mechanism-specific therapies in ACS.

## 7. OCT-Guided Strategy in Plaque Erosion: A New Frontier in Personalized ACS Management

In recent years, the management of ACS has evolved significantly, driven by a deeper understanding of its underlying pathophysiological mechanisms. Traditionally, ACS has been treated uniformly with an invasive strategy involving coronary stenting and DAPT, regardless of the specific plaque characteristics. However, emerging evidence has highlighted the distinct entity of PE, which differs from the classic PR in both morphology and clinical implications. This has sparked growing interest in whether a tailored, less invasive approach—focused on medical therapy alone—may be appropriate in selecting patients. Such a strategy could potentially minimize stent-related complications and instead promote natural endothelial healing, paving the way for a more personalized form of ACS management.

### 7.1. The EROSION Trial

Identifying PE in the catheterization laboratory is not merely academic; it has direct implications for patient management. Traditionally, the treatment for an ACS culprit lesion is to perform PCI with stent implantation, especially if there is a flow-limiting plaque or high-grade stenosis [48]. However, when OCT reveals a PE as the cause of ACS, especially if the underlying plaque is not severely obstructive, a more conservative, tailored strategy can be considered. Several studies have demonstrated that patients with PE tend to experience better clinical outcomes than those with PR, including a lower incidence of MACE during follow-up [49]. Building on this evidence, OCT-based investigations have shown that patients with OCT-confirmed PE, whether treated conservatively with dual antiplatelet therapy (DAPT) alone or with PCI, often remain asymptomatic for more than two years, regardless of stent implantation [50]. This evidence has supported the rationale that if there is no evidence of rupture or deep plaque cavity and the thrombus burden can be managed, the patient might be treated with intensive pharmacotherapy alone, avoiding the potential risks of stent implantation such as stent thrombosis (ST) or the need for long-term DAPT beyond what is needed for the ACS itself.

This concept was tested by the landmark EROSION study (Effective Anti-Thrombotic Therapy Without Stenting: Intravascular Optical Coherence Tomography-Based Management in Plaque Erosion) [51]. This study was a pivotal proof-of-concept trial that prospectively evaluated whether patients with OCT-confirmed PE could be safely treated with antiplatelet therapy alone. In this single-arm study, patients with less than 70% residual diameter stenosis and TIMI 3 flow were managed with aspirin and ticagrelor, without stent implantation. At 1-month follow-up, 85% of patients demonstrated >50% thrombus volume reduction, and 92.5% were free of MACE. These results challenged the prevailing dogma that all ACSs require stenting. Although this study has opened the door to a new therapeutic model, its results must be interpreted in light of several limitations: it was a small, single-center study without randomization. The 1-year follow-up of the EROSION study provided critical confirmation of the strategy’s mid-term safety [52]. Among the 53 patients who completed follow-up, 49 underwent repeat OCT imaging. Thrombus burden continued to decline between the 1-month and 1-year marks, and nearly half of the patients had no residual thrombus at all. Importantly, 92.5% of patients remained free from MACE. Only three patients required non-urgent revascularization due to exertional angina, and one experienced a non-cardiac bleeding event. The sustained thrombus regression, combined with the absence of recurrent ischemic events, strongly supports the feasibility of individualized treatment in patients with PE. This experience reinforces the concept that in selected cases, pharmacologic therapy alone may be sufficient to stabilize the culprit lesion and avoid stent-related complications. The strategy’s long-term implications were clarified in the four-year follow-up [53]. Of the 52 patients who completed follow-up (median 4.8 years), none experienced hard events such as death, MI, stroke, or heart failure. However, 11 patients (21%) underwent elective target lesion revascularization (TLR), mostly due to progressive angina or persistent stenosis. Strikingly, the best long-term outcomes were observed in patients who had shown significant thrombus resolution (>50%) at one month. Nearly all patients in the non-TLR group met this endpoint (95% vs. 45% in TLR group), underscoring the prognostic value of early thrombus dynamics. This emphasizes that early OCT-assessed thrombus dynamics may serve not only as a short-term marker of treatment efficacy but also as a long-term prognostic tool. The absence of hard events over nearly five years confirms the safety of a non-stenting approach in appropriately selected patients. However, the fact that one in five of the patients still required revascularization points to the necessity of careful monitoring and possibly refining selection criteria for conservative treatment.

While the EROSION trial provided important proof-of-concept evidence supporting a conservative, non-stent-based strategy in selected patients with OCT-confirmed PE, its applicability to higher-risk populations remains limited. The study excluded patients with significant left ventricular dysfunction (LVEF < 30%), renal impairment (creatinine > 2.0 mg/dL or end-stage kidney disease), hemodynamic or electrical instability, and those with reduced life expectancy. As such, individuals with advanced chronic kidney disease or frailty were not represented in the trial population. Nevertheless, patients with diabetes mellitus and prior ACS events were not explicitly excluded. The baseline population characteristics of the EROSION trial and its long-term follow-up cohorts report that 23% of patients had diabetes mellitus and 12% had a history of MI. These findings suggest that a stent-free approach may still be feasible in selected high-risk patients, although subgroup-specific outcomes were not reported. Further insight comes from a retrospective study by Hu et al. [54], which included 141 ACS patients with OCT-confirmed PR or PE and did not impose formal exclusion criteria. In this broader real-world population, a subset of erosion patients managed without stenting experienced no MACE at one year, further supporting the notion that individualized, imaging-guided management may be appropriate even in complex patients.

Despite these encouraging observations, robust prospective data in patients with diabetes, chronic kidney disease, or recurrent ischemic events are still lacking. Caution is warranted when extrapolating conservative strategies to such populations, which are often characterized by greater plaque complexity, systemic inflammation, and prothrombotic risk. Until further evidence becomes available, therapeutic decisions in these patients should be guided by careful clinical and imaging assessment on a case-by-case basis.

Finally, current evidence suggests that in PE, management decisions should not rely solely on angiographic severity or the presence of ACS but rather on lesion morphology, thrombus burden, and dynamic response to therapy. Early thrombus resolution, measurable with OCT, appears to be a strong marker of favorable outcome and may guide clinicians in determining who can safely avoid stent implantation. Looking ahead, the shift toward a personalized, pathophysiology-based approach to ACS treatment seems not only feasible but necessary. PE represents a biologically distinct entity from PR, characterized by endothelial denudation, hyaluronan-mediated inflammation, and neutrophil extracellular trap formation, suggesting that it may respond differently to therapies and carry different risks. For this reason, prediction tools that combine clinical, biochemical, and imaging markers are essential to identify erosion cases non-invasively and optimize their treatment. A future in which therapy is tailored to plaque phenotype, where only those with high-risk features or poor thrombus resolution receive stents, may reduce unnecessary interventions, limit long-term complications, and better align treatment with individual disease mechanisms.

### 7.2. Who Should Avoid a Stent? Patient Selection in Plaque Erosion Through the Lens of OCT Evidence

The clinical implications of the EROSION study are significant. The pioneering findings from this trial suggest that in selected ACS patients with OCT-confirmed PE, a strategy of intensive antithrombotic therapy (e.g., potent dual antiplatelets and sometimes short-term anticoagulation or glycoprotein IIb/IIIa inhibitors in certain protocols) can stabilize the lesion and achieve healing without the need for an immediate stent.

It points to a potential paradigm shift where a subset of ACSs—those due to PE—might be managed with a pharmacological approach, reserving stents only for those who truly need it. This personalized approach could reduce unnecessary stenting and its associated costs and complications. However, it is crucial to emphasize that this non-stenting strategy is not yet the standard of care for all PEs, and careful patient selection is key.

When OCT is performed during an ACS workup, its findings can directly inform a tailored treatment strategy. If OCT reveals PE, the clinician may consider a conservative, antithrombotic approach instead of immediate stenting. In practice, this means that if the culprit lesion has a residual stenosis that is not severe and there is no OCT evidence of rupture or deep plaque cavity, intensive medical therapy alone can be pursued. By contrast, if OCT confirms a PR or shows a high-grade stenosis compromising flow, standard management with PCI and stent implantation remains necessary to secure the artery [55].

In this setting risk stratification is critical in deciding which erosion patients can be safely managed without stenting. Both clinical and OCT-based criteria have emerged from studies like EROSION to guide this decision. Key favorable factors for conservative management include residual diameter stenosis less than 70%, TIMI 3 flow in the infarct-related artery, and a manageable thrombus burden without an extensive clot obstructing the lumen. Patients meeting these criteria—in particular, plaque erosion on OCT with a large residual lumen and TIMI 3 flow—have been shown to do well with medical therapy alone [51,52,53]. Pharmacologically, patients selected for conservative treatment must receive DAPT, typically with aspirin plus a potent P2Y12 inhibitor such as ticagrelor, as used in the EROSION protocol [51]. DAPT is generally maintained for at least 12 months, and some clinicians advocate prolonged therapy beyond one year in selected cases, considering the ongoing risk of thrombosis in a non-stented lesion. In the acute phase, intravenous glycoprotein IIb/IIIa inhibitors or short-term parenteral anticoagulation may be employed to accelerate thrombus dissolution.

To confirm therapeutic success and ensure lesion stabilization, a follow-up OCT at 1 to 3 months should be performed. In EROSION [51], follow-up imaging at one month revealed significant thrombus regression in most patients and, in some, complete thrombus resolution. This imaging-guided “watchful waiting” approach may allow a deferred, selective stenting decision only in cases of poor healing or persistent stenosis.

Emerging clinical evidence is rapidly shaping this personalized approach. The ongoing EROSION II trial is a multicenter study investigating OCT-guided deferral of stenting in a larger STEMI population, aiming to validate the criteria and safety of medical therapy in PE cases. Additionally, the recently published EROSION III trial [56] has provided robust data from a randomized perspective. In EROSION III, STEMI patients with TIMI 3 flow and <70% stenosis on initial angiography were randomized to OCT-guided therapy vs. standard care. The OCT arm used a “mechanism-based” strategy: if OCT-confirmed PE operators could opt to withhold stenting and treat with medications alone. Strikingly, OCT guidance led to a significantly lower rate of stent implantation: only 43.8% in the OCT arm vs. 58.8% in the angiography arm, indicating a 15% absolute reduction in stent use with OCT guidance. Moreover, patients who did receive stents under OCT guidance had better procedural results, with lower residual stenosis after implantation. Importantly, safety outcomes were comparable between the two groups, with no reinfarctions and similar rates of cardiac events after one year.

In this landscape, recent studies have further deepened the understanding of lesion behavior in non-stented erosion. Yin et al. used serial OCT to show that plaque healing is a common phenomenon in non-stented erosions, with more than half of patients developing evidence of healed plaques over time. Importantly, healing was more likely in those with larger thrombus burden and less severe baseline stenosis, suggesting that natural resolution can occur in favorable anatomical settings [57]. However, these findings are counterbalanced by the results of another study from the same group, which explored predictors of adverse events in non-stented erosion patients. Older age, greater area stenosis and higher thrombus burden were all associated with increased risk of MACE. When all three risk factors were present, the event rate exceeded 50%, indicating that a conservative approach may not be appropriate in these high-risk profiles [58].

In summary, the management of ACS caused by PE is transitioning from a uniform, device-based paradigm to a more nuanced and individualized strategy, grounded in intracoronary imaging and biological understanding. The EROSION study and its follow-ups have laid the foundation for this shift, and the future lies in refining predictive models and conducting prospective trials to validate non-stenting approaches in appropriately selected patients. Moreover, future randomized trials are needed to definitively confirm that an OCT-guided medical therapy approach is equivalent or superior to routine stenting in eligible erosion patients. If confirmed, this would herald a new era of truly personalized interventional cardiology, where the decision to stent or not is based on the mechanism of plaque destabilization rather than just the angiographic appearance.

### 7.3. Next-Generation Management of Plaque Erosion

Aside from the possibility of deferring stents, recognizing PE may also influence the choice of adjunctive therapies beyond DAPT. While optimal medical therapy (dual antiplatelets, high-intensity statin, etc.) and PCI with stent implantation remain the standard approach for ACS patients [37,48], there is considerable interest in identifying alternative treatment pathways for PE cases. Among the most actively explored options is the use of drug-coated balloons (DCBs).

These devices allow localized delivery of antiproliferative drugs (e.g., paclitaxel or sirolimus) to the vessel wall without leaving behind a permanent implant, potentially offering a means to treat certain erosion lesions with minimal mechanical intervention [59]. The use of DCBs in ACS has gained increasing attention in recent years, particularly as an alternative to stenting in selected patients. A foundational contribution in this field is the BASKET-SMALL 2 trial, a large randomized controlled study evaluating the safety and efficacy of DCBs versus drug-eluting stents in small-vessel CAD [60]. Among the 758 patients enrolled, nearly 30% presented with ACS, including STEMI and NSTE-ACS. Over both 1- and 3-year follow-ups, the trial found no significant difference in MACE between patients treated with DCBs or drug-eluting stents, indicating that DCBs are a viable and safe alternative in the ACS setting. Importantly, in ACS patients, the DCB strategy was associated with numerically lower rates of cardiac death and nonfatal myocardial infarction at 1 year compared to DES, although the study was not powered to detect superiority. These findings suggest that in certain ACS populations—particularly those with small vessels or high bleeding risk—the concept of “leaving nothing behind” with DCBs may offer both safety and clinical benefit.

Building on this groundwork, recent investigations have started to explore more pathophysiology-driven indications for DCB use, particularly in the context of PE. A notable example is the study by Yamamoto et al. [61], which specifically assessed the long-term outcomes of ACS patients treated with a DCB-only strategy based on OCT-defined plaque morphology. In this multicenter observational study of 127 patients, OCT imaging was used to classify culprit lesions into PE, PR, or CNs. The results were striking; patients with PE had significantly better outcomes, with a 3-year target lesion failure of just 7.5%, compared to 26.1% in PR and 43.5% in CN. What further distinguished this study was its demonstration of the prognostic value of residual thrombus burden after DCB treatment. Even among patients with PR, those with minimal thrombus left after the procedure had outcomes approaching those seen in the erosion group. This suggests that successful thrombus resolution—regardless of plaque type—may be a key determinant of long-term outcomes and that DCBs may be most appropriate in settings where effective thrombus clearance can be achieved. The Yamamoto study thus highlights a potential paradigm shift in the management of plaque erosion: in selected cases with favorable anatomical and thrombotic profiles, DCBs may represent a safe, effective, and stent-free option, especially when guided by high-resolution imaging such as OCT. These findings move the conversation beyond simple vessel size or bleeding risk, toward a more nuanced, mechanism-based approach to ACS treatment, tailoring therapy to the underlying pathophysiology of the lesion rather than applying a uniform stent-based strategy.

Alongside the development of new interventional devices, there is growing interest in enhancing plaque stabilization through systemic medical therapy, particularly in the context of plaque erosion. Since erosion is typically less lipid-rich and more platelet-driven compared to ruptured plaques, it may benefit particularly from interventions aimed at improving endothelial function and reducing subtle inflammation. High-intensity statins remain foundational not only for lipid lowering but also for their pleiotropic effects, including plaque stabilization and promotion of endothelial repair. In addition, angiotensin-converting enzyme (ACE) inhibitors or angiotensin receptor blockers (ARBs) should be prioritized, especially in patients with hypertension, diabetes, or impaired ventricular function, as these agents have shown endothelial benefits and may mitigate shear-stress-induced endothelial injury, which plays a role in erosion pathogenesis [55]. Looking to the future, anti-inflammatory therapies such as colchicine or interleukin-1β inhibitors may have a role in preventing recurrent erosion events, although their efficacy specifically in erosion has not yet been proven. These agents are being tested more broadly in post-ACS settings [62,63] and may one day be tailored to the biological subtype of plaque involved. Finally, the long-term outlook for patients with PE must include aggressive risk factor modification, with particular emphasis on smoking cessation. Smoking is strongly associated with endothelial dysfunction and is a well-recognized risk factor for erosion [9,64]. Continued tobacco exposure may not only hinder healing but also predispose to new erosion events. In addition, optimizing blood pressure, glycemic control, body weight, and adherence to lifestyle changes must remain a core component of secondary prevention strategies [37].

## 8. Conclusions and Future Perspectives

Plaque erosion has rapidly gained recognition as a major mechanism of ACS, now accounting for roughly 30–40% of cases. What was once considered a rare post-mortem finding is today frequently identified in living patients thanks to high-resolution OCT imaging. Erosion-prone patients tend to be younger, often female, and may lack classic risk factors aside from smoking, underlining a distinct clinical phenotype of ACS. Pathologically, erosion represents a different route to thrombosis than PR: it is driven by superficial endothelial injury and shear stress with a modest inflammatory component, in contrast to the intense inflammation and cap disruption of rupture. These differences have practical importance. Clinicians can now diagnose PE in the catheterization lab using OCT criteria, distinguishing it from PR with high confidence. Perhaps most exciting are the therapeutic implications: early studies like the EROSION trial indicate that a tailored, less invasive approach (intense pharmacotherapy without immediate stenting) can be effective in managing erosion-related ACS. This opens the door to personalized ACS management—treating the patient based on the underlying plaque pathology. Going forward, larger trials and long-term follow-ups will clarify which erosion patients can safely avoid stents and how best to promote healing of the endothelium. In parallel, research into biomarkers and non-invasive imaging may one day allow clinicians to suspect PE before invasive angiography, and novel therapies targeting the unique biology of erosion (such as anti-TLR2 or NET-inhibiting strategies) could emerge. In summary, PE is now established as a common cause of ACS with distinct characteristics. A thorough understanding of its epidemiology, identification by OCT, and optimal management strategies will enable cardiologists to improve patient outcomes through mechanism-guided therapy.

*“We shape our tools and thereafter our tools shape us”*. This insight from Marshall McLuhan resonates now more than ever in cardiovascular medicine. The evolution of OCT—from a mere diagnostic advancement to a driver of paradigm shifts—has profoundly altered our understanding and management of acute coronary syndromes. By illuminating the role of PE as a distinct and prevalent mechanism, OCT has enabled clinicians to move beyond traditional models rooted solely in PR, ushering in an era of mechanism-guided, personalized therapy. As our tools become more precise, so too must our thinking: reshaped by what we can now see and redefined by what we can now do. In this way, the future of ACS care will not be dictated solely by the presence of a lesion but by a deeper understanding of its nature—and by the thoughtful application of the tools that allow us to see it.

## Data Availability

All data generated or analyzed during this study are included in this published article. Further inquiries should be directed to the corresponding author.

## Abbreviations

The following abbreviations are used in this manuscript:

ACS	Acute coronary syndrome
PR	Plaque rupture
PE	Plaque erosion
MI	Myocardial infarction
OCT	Optical coherence tomography
CAD	Coronary artery disease
NSTE-ACS	Non-ST elevation ACS
CN	Calcified nodule
VSMC	Vascular smooth muscle cells
AMI	Acute myocardial infarction
TCFA	Thin-cap fibroatheroma
CCs	Cholesterol crystals
IVI	Intravascular imaging
STEMI	ST-elevation myocardial infarction
TLR2	Toll-like receptor 2
NET	Neutrophil extracellular traps
MPO	Myeloperoxidase
RFC	Rupture fibrous cap
IFC	Intact fibrous cap
MMP	Matrix metalloproteinase
MACE	Major adverse cardiac events
EndMT	Endothelial-to-mesenchymal transition
QCA	Quantitative coronary angiography
IVUS	Intravascular ultrasound
NIRS	Near-infrared spectroscopy
CCTA	Coronary computed tomography angiography
LAD	Left anterior descending artery
MLA	Minimal lumen area
HD-IVUS	High-definition intravascular ultrasound
VH-IVUS	Virtual histology intravascular ultrasound
LCBI	Lipid core burden index
CT	Computed tomography
PVAT	Perivascular adipose tissue
FAI	Fatty attenuation index
PCCT	Photon-counting computed tomography
TLR	Target lesion revascularization
DCB	Drug-coated balloon
ACE	Angiotensin-converting enzyme
ARBs	Angiotensin receptor blockers
